# Dual role of the integrated stress response in medulloblastoma tumorigenesis

**DOI:** 10.18632/oncotarget.11873

**Published:** 2016-09-06

**Authors:** Sarrabeth Stone, Yeung Ho, Xiting Li, Stephanie Jamison, Heather P. Harding, David Ron, Wensheng Lin

**Affiliations:** ^1^ Department of Neuroscience, University of Minnesota, Minneapolis, Minnesota, United States; ^2^ Institute for Translational Neuroscience, University of Minnesota, Minneapolis, Minnesota, United States; ^3^ Masonic Cancer Center, University of Minnesota, Minneapolis, Minnesota, United States; ^4^ Department of Periodontics, Guanghua School of Stomatology, Hospital of Stomatology, Sun Yat-sen University, Guangzhou, Guangdong, China; ^5^ Cambridge Institute of Medical Research, University of Cambridge, Cambridge, United Kingdom

**Keywords:** medulloblastoma, integrated stress response, ER stress, GADD34, tumorigenesis

## Abstract

In response to endoplasmic reticulum (ER) stress, activation of pancreatic ER kinase (PERK) coordinates an adaptive program known as the integrated stress response (ISR) by phosphorylating translation initiation factor 2α (eIF2α). Phosphorylated eIF2α is quickly dephosphorylated by the protein phosphatase 1 and growth arrest and DNA damage 34 (GADD34) complex. Data indicate that the ISR can either promote or suppress tumor development. Our previous studies showed that the ISR is activated in medulloblastoma in both human patients and animal models, and that the decreased ISR via PERK heterozygous deficiency attenuates medulloblastoma formation in *Patched1* heterozygous deficient (*Ptch1*+/−) mice by enhancing apoptosis of pre-malignant granule cell precursors (GCPs) during cell transformation. We showed here that GADD34 heterozygous mutation moderately enhanced the ISR and noticeably increased the incidence of medulloblastoma in adult *Ptch1*+/− mice. Surprisingly, GADD34 homozygous mutation strongly enhanced the ISR, but significantly decreased the incidence of medulloblastoma in adult *Ptch1*+/− mice. Intriguingly, GADD34 homozygous mutation significantly enhanced pre-malignant GCP apoptosis in cerebellar hyperplastic lesions and reduced the lesion numbers in young *Ptch1*+/− mice. Nevertheless, neither GADD34 heterozygous mutation nor GADD34 homozygous mutation had a significant effect on medulloblastoma cells in adult *Ptch1*+/− mice. Collectively, these data imply the dual role of the ISR, promoting and inhibiting, in medulloblastoma tumorigenesis by regulating apoptosis of pre-malignant GCPs during the course of malignant transformation.

## INTRODUCTION

**Figure 1:**
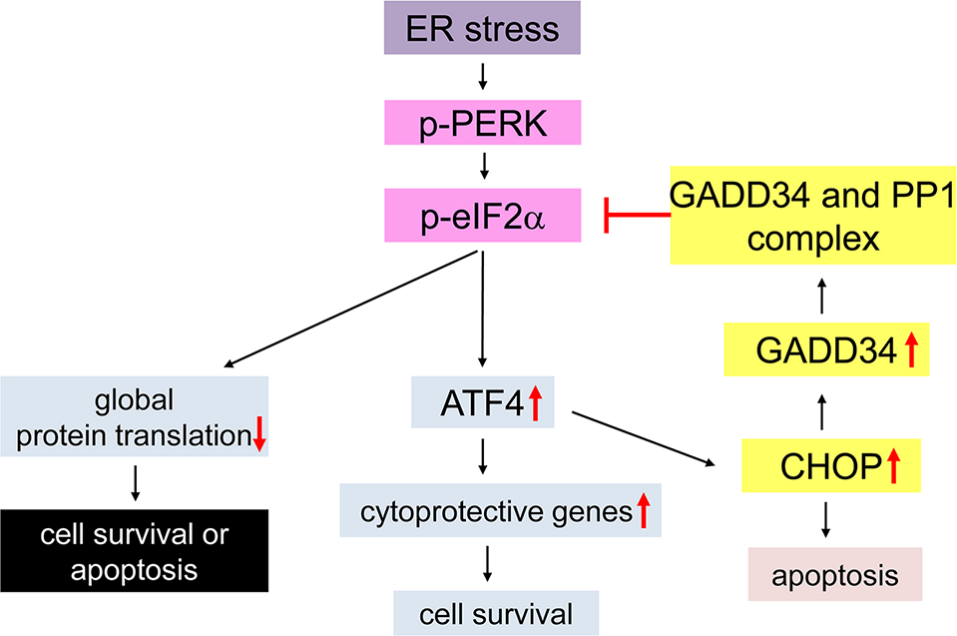
Schematic diagram of the PERK-mediated ISR Under conditions of ER stress the PERK protein becomes activated by homodimerization and autophosphorylation (p-PERK). p-PERK phosphorylates eIF2α, and results in inhibition of global protein translation and induction of cytoprotective genes by preferentially stimulating translation of ATF4. ATF4 also enhances the expression of CHOP, which negatively regulates p-eIF2α level through induction of GADD34 that binds PP1 and dephosphorylates p-eIF2α. The moderate ISR promotes cell survival by modestly inhibiting protein translation and enhancing the expression of certain cytoprotective genes. In contrast, the strong ISR leads to cell apoptosis by strongly inhibiting protein translation and/or inducing CHOP expression.

Activation of pancreatic endoplasmic reticulum kinase (PERK), in response to endoplasmic reticulum (ER) stress, coordinates an adaptive program known as the integrated stress response (ISR, Figure 1) by phosphorylating the α subunit of the eukaryotic translation initiation factor 2 (eIF2α) [1, 2, 3]. Phosphorylation of eIF2α inhibits global protein translation but stimulates the expression of certain stress-induced genes through induction of the transcription factor ATF4. On the other hand, phosphorylated eIF2α (p-eIF2α) is quickly dephosphorylated by the protein phosphatase 1 (PP1) and growth arrest and DNA damage 34 (GADD34) complex [4, 5], allowing cells to recover from inhibition of global protein biosynthesis. Interestingly, induction of ATF4 by p-eIF2α stimulates the expression of the transcription factor CAATT enhancer binding protein homologous protein (CHOP), and results in upregulation of GADD34, which forms a tight autofeedback loop regulating activity of the ISR [6, 7, 8].

Several lines of evidence have suggested that the PERK-mediated ISR plays an important role in tumor growth and invasion, including regulating tumor cell viability, tumor cell invasion, and angiogenesis [9, 10, 11]. Interestingly, recent studies showed that the ISR is also involved in the process of malignant transformation by regulating cell apoptosis [12, 13, 14]. However, the effects of the ISR on tumor development are controversial [15, 16, 17]. Some reports showed that the ISR attenuates cell apoptosis during malignant transformation and/or after malignant transformation, resulting in enhanced tumor development [10, 12]. In contrast, other reports showed that the ISR elevates cell apoptosis during malignant transformation and/or after malignant transformation, resulting in attenuated tumor development [13, 18].

Medulloblastoma can be classified into four major molecular subgroups based on the molecular profiling: sonic hedgehog (SHH) subgroup, WNT subgroup, subgroup 3, and subgroup 4 [19, 20]. Mice heterozygous for *Patched1* (*Ptch1+/−*), a SHH receptor, are regarded as the best animal model to study the SHH subgroup of medulloblastoma [21, 22]. The development of medulloblastoma in *Ptch1+/−* mice exhibits distinct steps of progression [23, 24, 25]. These mice display hyperplastic lesions of pre-malignant granule cell precursors (GCPs) in the external granular layer (EGL) of the cerebellum starting at the age of 3 weeks, and the lesions persist as late as 6 weeks of age. While a few hyperplastic lesions undergo cell transformation and progress to malignancy, the majority of the lesions regress over time.

Recent studies showed that the PERK-mediated ISR participates in medulloblastoma development [23, 26, 27]. Activation of PERK signaling has been observed in pre-malignant GCPs in young *Ptch1+/−* mice and in medulloblastoma cells in human patients and animal models [27]. Importantly, our previous study showed that PERK haploinsufficiency reduces the incidence of medulloblastoma in *Ptch1+/−* mice. Intriguingly, PERK haploinsufficiency enhances apoptosis of pre-malignant GCPs in young *Ptch1+/−* mice, but has no significant effect on medulloblastoma cells in adult mice [27]. In this study, we sought to better understand the effects of the ISR on the development of medulloblastoma in *Ptch1+/−* mice by exploiting GADD34 mutant mice. We found that GADD34 heterozygous mutation noticeably increased the incidence of medulloblastoma in *Ptch1+/−* mice. Surprisingly, GADD34 homozygous mutation significantly decreased the incidence of medulloblastoma in *Ptch1+/−* mice, which was associated with enhanced apoptosis of pre-malignant GCPs in young mice. Additionally, GADD34 mutation, either heterozygous or homozygous, did not affect medulloblastoma cells in adult *Ptch1+/−* mice. Collectively, these data suggest a dual role of the ISR, promoting and inhibiting, in medulloblastoma tumorigenesis.

## RESULTS

### Dual role of the ISR in medulloblastoma formation in *Ptch1*+/− mice

*Ptch1+/−* mice develop symptomatic medulloblastoma typically between the ages of 2 and 8 months [22, 27, 28]. Our previous study demonstrated activation of the PERK-mediated ISR in medulloblastoma cells in adult *Ptch1+/−* mice and in pre-malignant GCPs in young mice [27]. We also showed that GADD34 is upregulated in medulloblastoma in *Ptch1+/−* mice [27]. It is well documented that inhibition of GADD34, via either genetic or pharmacological approaches, elevates the ISR during ER stress [29, 30]. *Gadd34* homozygous mutant (*Gadd34−/−*) mice appear healthy and do not display any abnormalities in the CNS under normal conditions [7, 26, 29]. Moreover, a report showed that *Gadd34* heterozygous mutant (*Gadd34+/−*) mice are healthy and display evidence of haploinsufficiency [31]. To determine the effects of the enhanced ISR on medulloblastoma development, *Ptch1+/−* mice were crossed with *Gadd34−/−* mice, and then the resulting progeny were intercrossed to obtain *Ptch1+/−*; *Gadd34 wild type* (*Gadd34+/+*) mice, *Ptch1+/−*; *Gadd34+/−* mice, and *Ptch1+/−; Gadd34−/−* mice. We monitored these mice daily up to 8 months to detect medulloblastoma phenotypes, including ataxia, decreased movement, poor grooming, and doming of the skull [21, 22]. We verified medulloblastoma formation in symptomatic mice by gross examination, namely determining enlargement of the skull and cerebellum, and by hematoxylin and eosin (H&E) staining.

**Figure 2:**
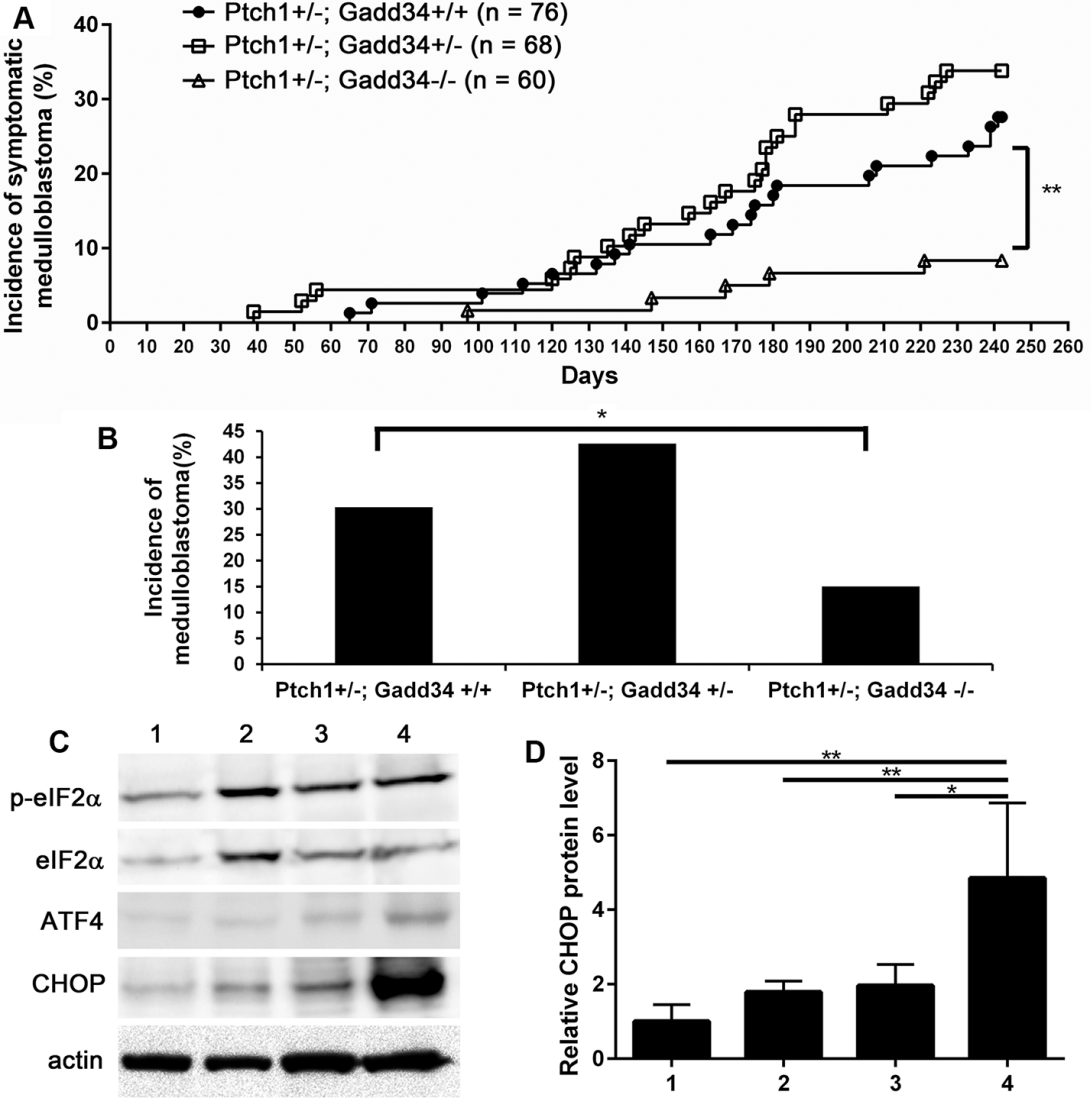
GADD34 mutation influenced medulloblastoma formation in *Ptch1+/−* mice (**A**) The percentage of mice that developed symptomatic medulloblastoma by the age of 8 months. (**B**) The percentage of mice that developed medulloblastoma by the age of 8 months. *Ptch1+/−; Gadd34+/+* mice, *n* = 76 animals; *Ptch1+/−; Gadd34+/−* mice, *n* = 68 animals; *Ptch1+/−; Gadd34−/−* mice, *n* = 60 animal. (**C**, **D**) Western blot analysis showed that GADD34 heterozygous mutation did not change p-eIF2α level, modestly elevated ATF4 level, and moderately increased CHOP level in the cerebellum of adult *Ptch1+/−* mice with medulloblastoma, and that GADD34 homozygous mutation did not change p-eIF2α level, moderately elevated ATF4 level, and strongly increased CHOP level in the cerebellum of adult *Ptch1+/−* mice with medulloblastoma. 1, wild type mice; 2, *Ptch1+/−; Gadd34+/+* mice; 3, *Ptch1+/−; Gadd34+/−* mice; 4, *Ptch1+/−; Gadd34−/−* mice. *N* = 4 animals. Error bars represent SD, ^**^P <0.01, ^*^P <0.05.

Our previous study showed that PERK heterozygous deficiency reduces the incidence of medulloblastoma in *Ptch1+/−* mice [27]. In agreement with this previous study, the frequency of symptomatic medulloblastoma in *Ptch1+/−; Gadd34+/−* mice was noticeably increased compared to *Ptch1+/−; Gadd34+/+* mice (Figure 2A). We further performed necropsy on all 8-month-old asymptomatic mice. Gross examination and H&E staining revealed that 2 out of 55 asymptomatic *Ptch1+/−*; *Gadd34+/+* mice and 6 out of 45 asymptomatic *Ptch1+/−; Gadd34+/−* mice developed medulloblastoma, respectively. Thus, GADD34 heterozygous mutation noticeably increased the incidence of medulloblastoma in *Ptch1+/−* mice (29 out of 68 *Ptch1+/−; GADD34+/−* mice vs 23 out of 76 *Ptch1+/−; GADD34+/+* mice, Figure 2B). Western blot analysis showed that GADD34 heterozygous mutation did not change p-eIF2α level, modestly elevated ATF4 level, and moderately increased CHOP level in medulloblastoma in *Ptch1+/−* mice (Figure 2C, 2D). It is known that elevation of p-eIF2α level in ER-stressed cells is transient and highly dynamic; however, elevation of CHOP level is relatively steady and long-lasting [8, 32]. The moderate elevation of CHOP level indicates moderately enhanced ISR in medulloblastoma in *Ptch1+/−; Gadd34+/−* mice compared to *Ptch1+/−; Gadd34+/+* mice. Taken together, these results suggest GADD34 heterozygous mutation results in the noticeable increase of medulloblastoma incidence in *Ptch1+/−* mice by moderately enhancing the ISR.

Western blot analysis showed that GADD34 homozygous mutation did not alter the level of p-eIF2α and moderately elevated the level of ATF4 in medulloblastoma in *Ptch1+/−* mice (Figure 2C). Importantly, the level of CHOP was strongly increased in medulloblastoma in *Ptch1+/−; Gadd34−/−* mice compared to *Ptch1+/−; Gadd34+/+* mice (Figure 2C, 2D), suggesting that GADD34 homozygous mutation strongly elevates the ISR in medulloblastoma in *Ptch1+/−* mice. Surprisingly, the frequency of symptomatic medulloblastoma in *Ptch1+/−; Gadd34−/−* mice was significantly decreased compared to *Ptch1+/−; Gadd34+/+* mice (Figure 2A). Moreover, gross examination and H&E staining revealed that 4 out of 55 asymptomatic *Ptch1+/−; Gadd34−/−* mice developed medulloblastoma. Thus, GADD34 homozygous mutation significantly reduced the incidence of medulloblastoma in *Ptch1+/−* mice (9 out of 60 *Ptch1+/−; Gadd34−/−* mice vs 23 out of 76 *Ptch1+/−; Gadd34+/+* mice, Figure 2B). Collectively, these results showed GADD34 homozygous mutation strongly enhances the ISR and results in the significant decrease of medulloblastoma incidence in *Ptch1+/−* mice.

### GADD34 mutation did not affect cell proliferation, cell apoptosis, or angiogenesis in medulloblastoma in adult *Ptch1*+/− mice

**Figure 3:**
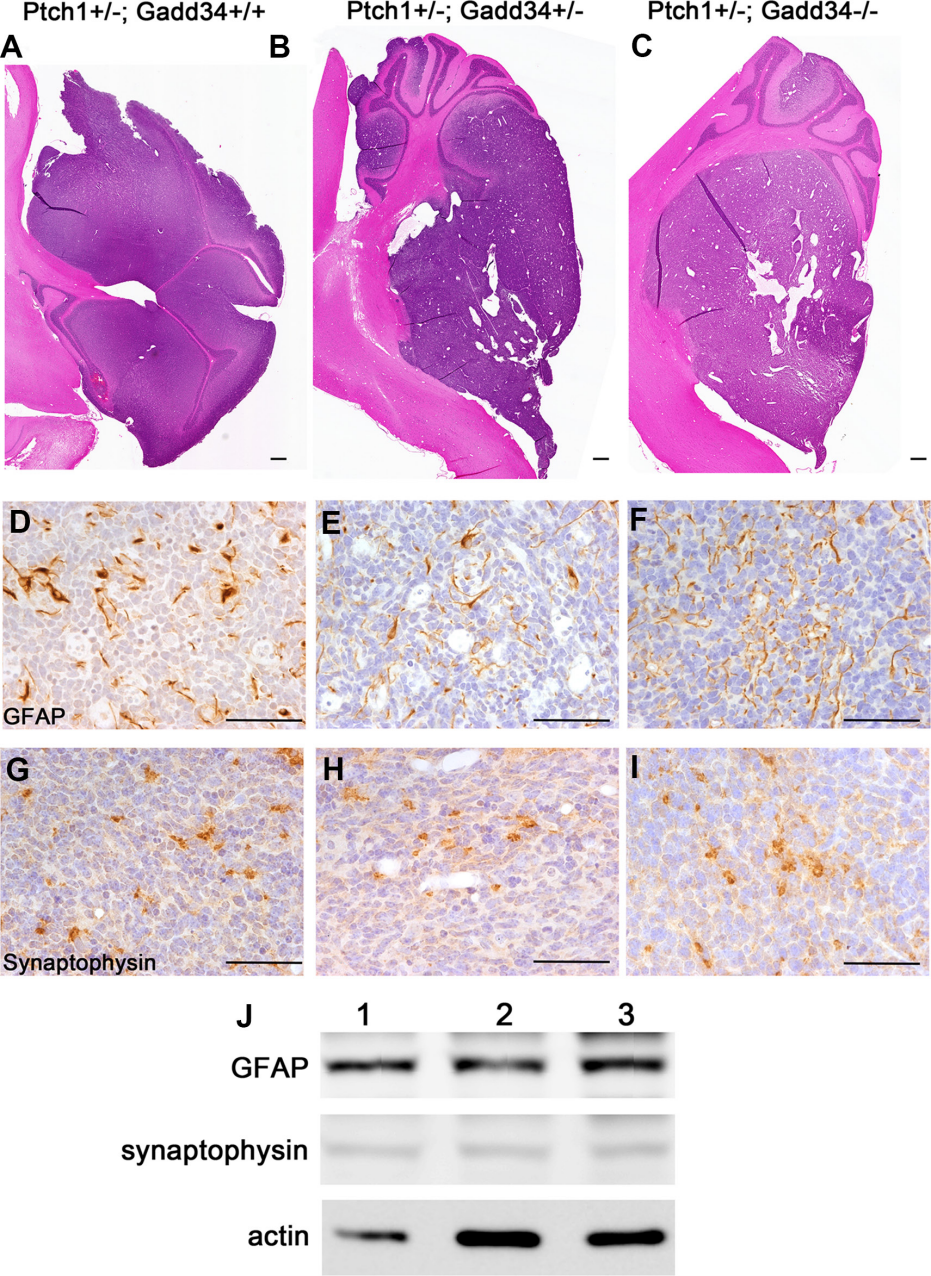
GADD34 mutation did not affect the morphology or differentiation of medulloblastoma in adult *Ptch1+/−* mice (**A**, **B**, **C**) H&E staining showed that GADD34 mutation, either heterozygous or homozygous, did not change the morphology of medulloblastoma in adult *Ptch1+/−* mice. (**D**, **E**, **F**) GFAP IHC showed that GADD34 mutation, either heterozygous or homozygous, did not change the expression pattern of GFAP in medulloblastoma in adult *Ptch1+/−* mice. (**G**, **H**, **I**) Synaptophysin IHC showed that GADD34 mutation, either heterozygous or homozygous, did not change the expression pattern of synaptophysin in medulloblastoma in adult *Ptch1+/−* mice. (**J**) Western blot analysis showed that GADD34 mutation, either heterozygous or homozygous, did not change the protein level of GFAP or synaptophysin in the cerebellum of adult *Ptch1+/−* mice with medulloblastoma. 1, *Ptch1+/−; Gadd34+/+* mice; 2, *Ptch1+/−; Gadd34+/−* mice; 3, *Ptch1+/−; Gadd34−/−* mice. *N* = 4 animals. Scale bars: A–C, 1000 μm; D–I, 50 μm.

**Figure 4:**
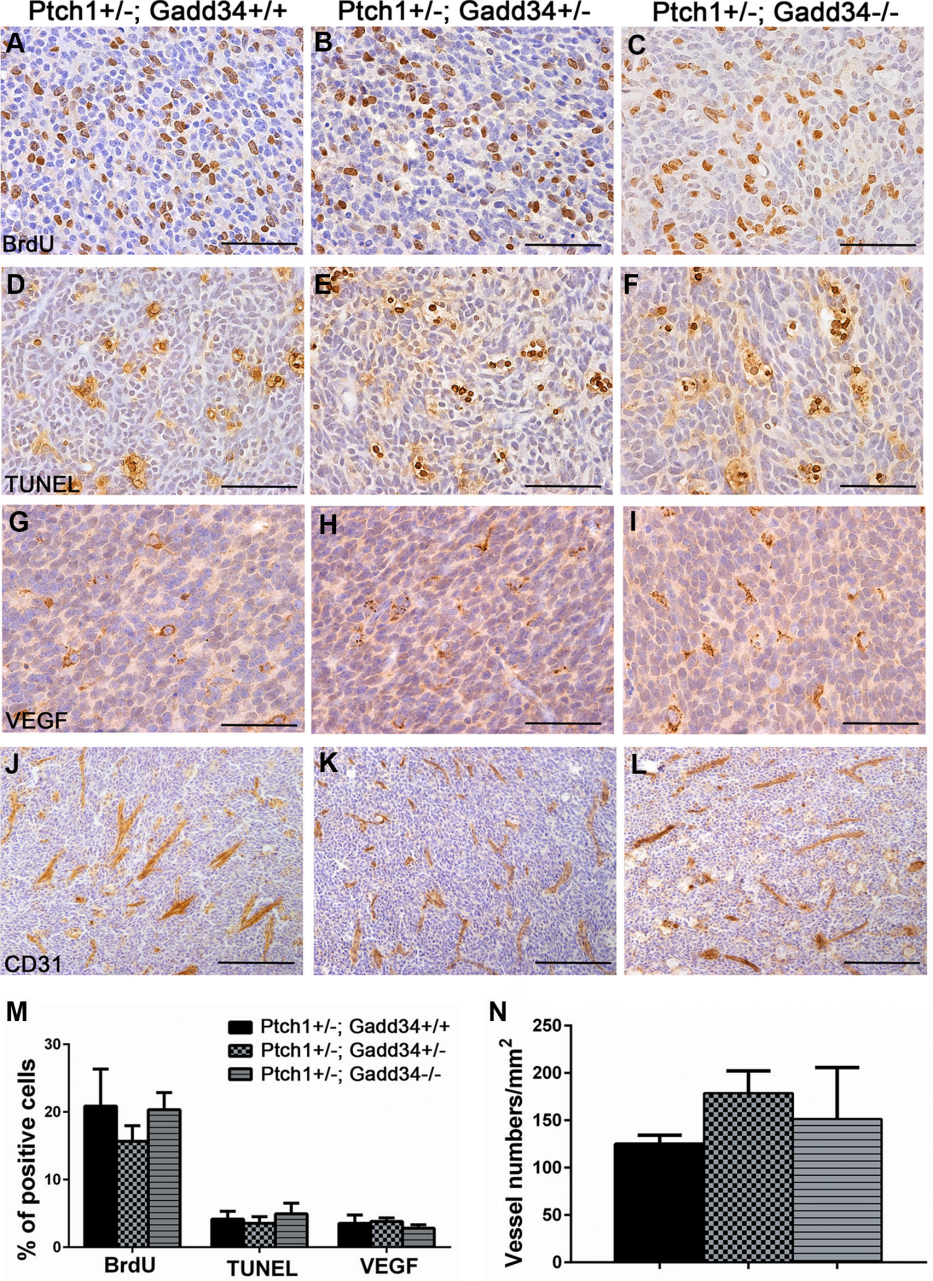
GADD34 mutation did not affect cell proliferation, cell apoptosis or angiogenesis in medulloblastoma in adult *Ptch1+/−* mice (**A**, **B**, **C**, **M**) BrdU labeling revealed comparable number of proliferating cells in medulloblastoma in adult *Ptch1+/−; Gadd34+/+* mice, *Ptch1+/−; Gadd34+/−* mice, and *Ptch1+/−; Gadd34−/−* mice. (**D**, **E**, **F**, **M**) TUNEL staining revealed comparable number of apoptotic cells in medulloblastoma in adult *Ptch1+/−; Gadd34+/+* mice, *Ptch1+/−; Gadd34+/−* mice, and *Ptch1+/−; Gadd34−/−* mice. (**G**, **H**, **I**, **M**) VEGF-A IHC revealed comparable number of VEGF-A positive cells in medulloblastoma in adult *Ptch1+/−; Gadd34+/+* mice, *Ptch1+/−; Gadd34+/−* mice, and *Ptch1+/−; Gadd34−/−* mice. (**J**, **K**, **L**, **N**) CD31 IHC revealed comparable number of blood vessels in medulloblastoma in adult *Ptch1+/−; Gadd34+/+* mice, *Ptch1+/−; Gadd34+/−* mice, and *Ptch1+/−; Gadd34−/−* mice. *N* = 4 animals. Error bars represent SD. Scale bars: (A–I), 50 μm; (J–L), 100 μm.

Our previous study showed that the decreased ISR via PERK heterozygous deficiency has no effect on cell proliferation, cell apoptosis, or angiogenesis in medulloblastoma in adult *Ptch1+/−* mice [27]. We determined the effects of the enhanced ISR on medulloblastoma in adult *Ptch1+/−* mice. H&E staining showed that the morphology of medulloblastoma in both *Ptch1+/−; Gadd34+/−* mice and *Ptch1+/−; Gadd34−/−* mice was comparable with *Ptch1+/−; Gadd34+/+* mice (Figure 3A–3C). Immunohistochemistry (IHC) for glial fibrillary acidic protein (GFAP) and synaptophysin showed that the expression pattern of both GFAP and synaptophysin in medulloblastoma in both *Ptch1+/−*; *Gadd34+/−* mice and *Ptch1+/−*;*Gadd34−/−* mice was comparable with *Ptch1+/−*; *Gadd34+/+* mice (Figure 3D–3I). Western blot analysis showed the protein levels of GFAP and synaptophysin were comparable in medulloblastoma in these three groups of mice (Figure 3J). Bromodeoxyuridine (BrdU) labeling (Figure 4A–4C, 4M) and proliferating cell nuclear antigen (PCNA) IHC (data not shown) showed that GADD34 mutation, either heterozygous or homozygous, did not significantly affect the number of proliferating cells in medulloblastoma in *Ptch1+/−* mice. Moreover, terminal deoxynucleotidyl transferase-mediated biotinylated UTP nick end labeling (TUNEL) staining showed that GADD34 mutation, either heterozygous or homozygous, did not significantly alter cell apoptosis in medulloblastoma in *Ptch1+/−* mice (Figure 4D–4F, 4M). Several studies showed that the ISR stimulates angiogenesis in tumors, including medulloblastoma, by enhancing the expression of vascular endothelial growth factor A (VEGF-A) [26, 33]. However, VEGF-A IHC showed that GADD34 mutation, either heterozygous or homozygous, had no significant effect on VEGF-A expression in medulloblastoma in *Ptch1+/−* mice (Figure 4G–4I, 4M), and CD31 IHC showed that GADD34 mutation, either heterozygous or homozygous, did not significantly affect angiogenesis in this tumor (Figure 4J–4L, 4N). Collectively, these data suggest that GADD34 mutation, either heterozygous or homozygous, has a minimal effect on cell proliferation, cell apoptosis, and angiogenesis in medulloblastoma in adult *Ptch1+/−* mice. Thus, it is unlikely that GADD34 mutation influences medulloblastoma formation in *Ptch1+/−* mice through its effects on tumor cells.

### GADD34 homozygous mutation increased pre-malignant GCPs apoptosis in the cerebellum of young *Ptch1* +/− mice

Medulloblastoma arises from GCPs in the cerebellar EGL of *Ptch1+/−* mice and exhibits distinct steps of progression [23, 24, 25]. GCPs proliferate, differentiate, migrate to the internal granule layer, and become mature granule neurons during early postnatal development. There are no GCPs in the EGL of normal mice at the age of 3 weeks [34]. Nevertheless, *Ptch1+/−* mice display hyperplastic lesions of pre-malignant GCPs in the cerebellar EGL starting at the age of 3 weeks, and the lesions persist as late as 6 weeks of age [23, 25, 35]. The majority of these hyperplastic lesions regress over time; however, a few hyperplastic lesions undergo cell transformation and progress to malignancy [25, 36]. Our previous study showed that the decreased ISR via PERK heterozygous deficiency enhances pre-malignant GCP apoptosis, resulting in the reduction of medulloblastoma incidence in *Ptch1+/−* mice [27]. We further determined the actions of the enhanced ISR in pre-malignant GCPs in young *Ptch1+/−* mice.

**Figure 5:**
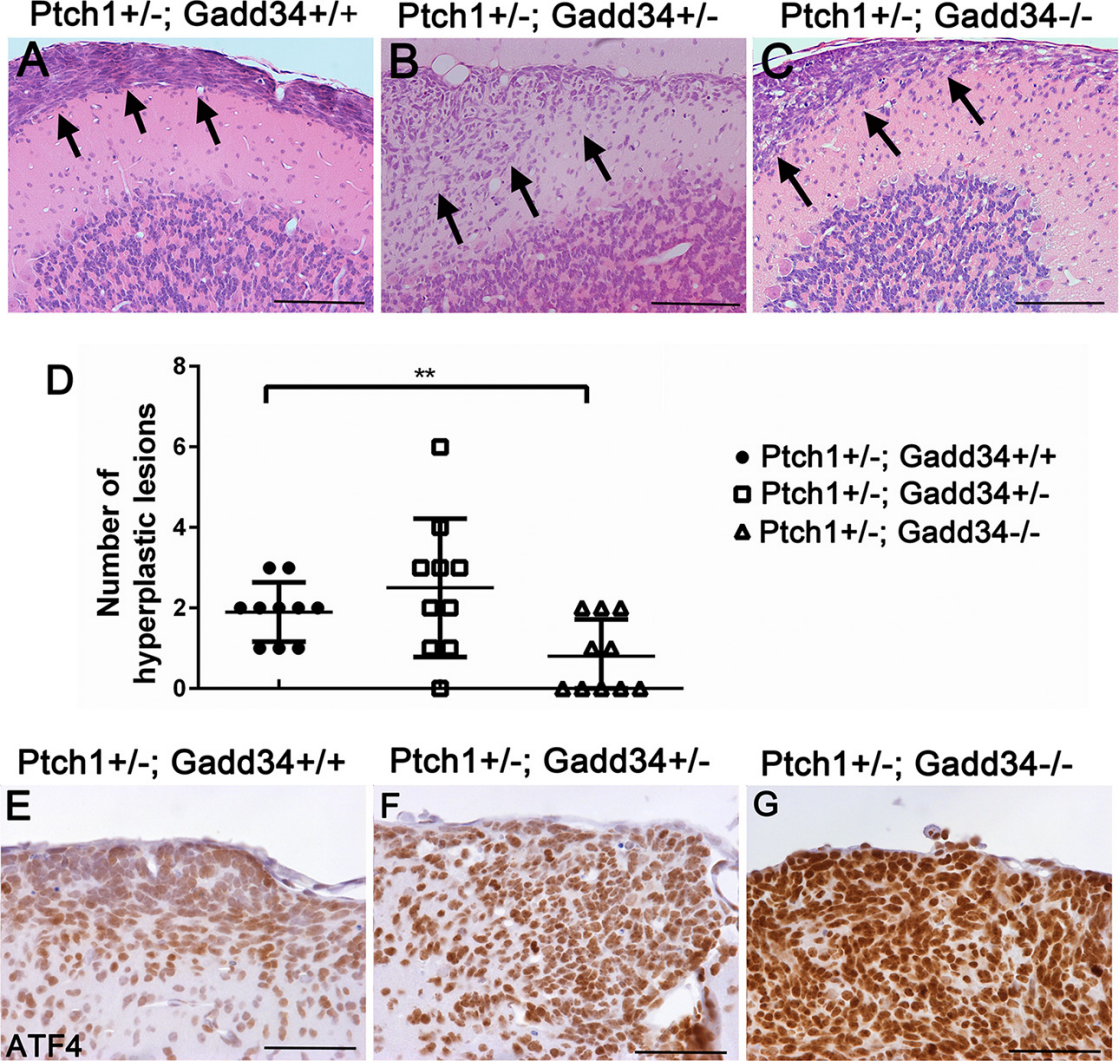
GADD34 mutation altered the number of cerebellar hyperplastic lesions in young *Ptch1+/−* mice (**A**, **B**, **C**) H&E staining revealed a hyperplastic lesion (arrows) in the cerebellum of 6-week-old *Ptch1+/−; Gadd34+/+* mice, *Ptch1+/−; Gadd34+/−* mice, and *Ptch1+/−; Gadd34−/−* mice. (**D**) The scatter plot showed that GADD34 heterozygous mutation slightly increased the number of hyperplastic lesions in the cerebellum of 6-week-old *Ptch1+/−* mice, and that GADD34 homozygous mutation significantly reduced the number of hyperplastic lesions in these mice. (**E**, **F**, **G**) ATF4 IHC showed that the level of ATF4 was moderately increased in hyperplastic lesions in the cerebellum of 6-week-old *Ptch1+/−; Gadd34+/−* mice and strongly increased *Ptch1+/−; Gadd34−/−* mice, compared to *Ptch1+/−; Gadd34+/+* mice. *N* = 10 animals. Error bars represent SD, ^**^P <0.01. Scale bars: (A–C), 200 μm; (E–G) 50 μm.

We serially sectioned whole paraffin-embedded half-cerebellum of 6-week-old mice, each half-cerebellum yielded ~ 210 5-μm-thick sections. Every tenth section in the series was either stained by H&E or immunostained by the NeuN antibody or PCNA antibody. The hyperplastic lesions (Figure 5A–5C), which contained greater than 10 pre-malignant GCPs that were negative for NeuN (a marker of mature neurons) and positive for PCNA were counted as described in our previous paper [27]. We found that the number of hyperplastic lesions in the cerebellum of 6-week-old *Ptch1+/−; Gadd34+/−* mice was slightly increased as compared to *Ptch1+/−; Gadd34+/+* mice (Figure 5D). Interestingly, the number of hyperplastic lesions in the cerebellum of 6-week-old *Ptch1+/−; Gadd34−/−* mice was significantly reduced as compared to *Ptch1+/−; Gadd34+/+* mice (Figure 5D).

**Figure 6:**
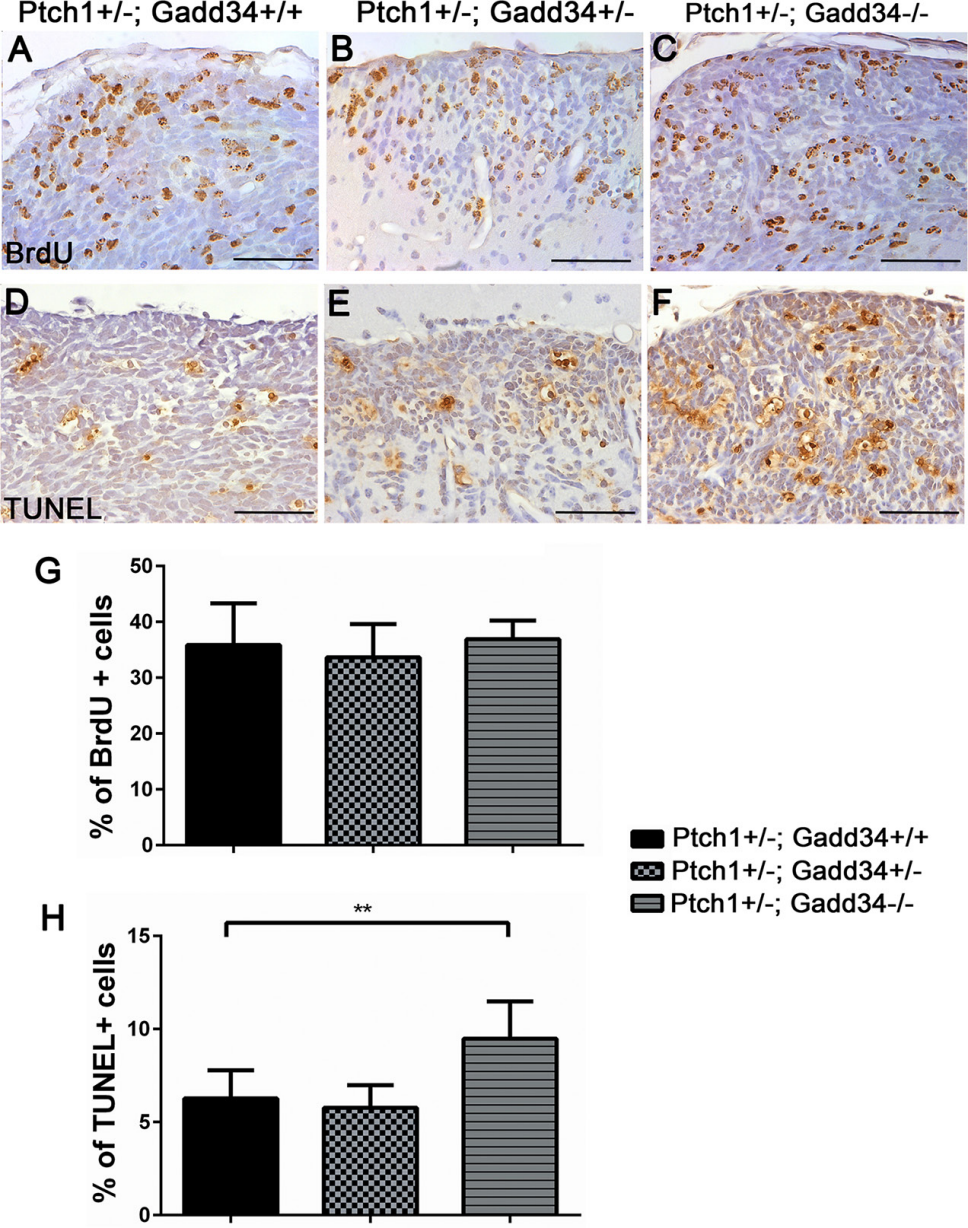
GADD34 homozygous mutation enhanced apoptosis of pre-malignant GCPs in hyperplastic lesions in the cerebellum of young Ptch1+/− mice (**A**, **B**, **C**, **G**) BrdU labeling revealed comparable number of proliferating cells in hyperplastic lesions in the cerebellum of 6-week-old *Ptch1+/−; Gadd34+/+* mice, *Ptch1+/−; Gadd34+/−* mice, and *Ptch1+/−; Gadd34−/−* mice. (**D**, **E**, **F**, **H**) TUNEL staining revealed significantly increased number of apoptotic cells in diffuse hyperplastic lesions in 6-week-old *Ptch1+/−; Gadd34−/−* mice compared to *Ptch1+/−; Gadd34+/+* mice. However, GADD34 heterozygous mutation did not alter the number of apoptotic pre-malignant GCPs in hyperplastic lesions in *Ptch1+/−* mice. *N* = 4 animals. Error bars represent SD, ^**^P < 0.01. Scale bars: (A–B), 50 μm.

ATF4 IHC showed that immunoreactivity of ATF4 was noticeably increased in hyperplastic lesions in 6-week-old *Ptch1+/−; Gadd34+/−* mice as compared to *Ptch1+/−; Gadd34+/+* mice (Figure 5E, 5F), and that the level of ATF4 was further increased in hyperplastic lesions in *Ptch1+/−; Gadd34*−/−mice (Figure 5G). BrdU labeling (Figure 6A–6C, 6G) showed that GADD34 mutation, either heterozygous or homozygous, did not significantly alter the rate of cell proliferation in hyperplastic lesions in 6-week-old *Ptch1+/−* mice. Importantly, TUNEL labeling showed that GADD34 homozygous mutation significantly increased the number of apoptotic cells in hyperplastic lesions in 6-week-old *Ptch1+/−* mice (Figure 6D, 6F, 6H). Nevertheless, GADD34 heterozygous mutation did not significantly change the number of apoptotic cells in hyperplastic lesions in 6-week-old *Ptch1+/−* mice (Figure 6D, 6E, 6H). Taken together, our data suggest that the strongly enhanced ISR via GADD34 homozygous mutation significantly promotes pre-malignant GCP apoptosis and reduces the number of hyperplastic lesions in young *Ptch1+/−* mice, resulting in the significantly decreased incidence of medulloblastoma in adult *Ptch1+/−* mice. Nevertheless, the moderately enhanced ISR via GADD34 heterozygous mutation slightly increases the number of hyperplastic lesions in young *Ptch1+/−* mice and leads to the noticeably elevated incidence of medulloblastoma in adult *Ptch1+/−* mice.

## DISCUSSION

Evidence is emerging that the PERK-mediated ISR can either promote or suppress tumorigenesis by regulating cell apoptosis during the course of malignant transformation [12, 13, 14, 17]. Using PERK heterozygous deficient mice, our previous study showed that the decreased ISR reduces the incidence of medulloblastoma in *Ptch1+/−* mice by enhancing pre-malignant GCP apoptosis during cell transformation [27]. In accordance with this previous study, we showed here that the moderately enhanced ISR via GADD34 heterozygous mutation slightly increased the number of hyperplastic lesions in young *Ptch1+/−* mice and noticeably increased the incidence of medulloblastoma in *Ptch1+/−* mice. Nevertheless, we also found that the strongly enhanced ISR via GADD34 homozygous mutation significantly increased pre-malignant GCP apoptosis in hyperplastic lesions and reduced the lesion numbers in young *Ptch1+/−* mice, resulting in the significant reduction of medulloblastoma incidence in adult *Ptch1+/−* mice. Interestingly, neither GADD34 heterozygous mutation nor GADD34 homozygous mutation had an effect on medulloblastoma cells in adult *Ptch1+/−* mice. Taken together, these findings suggest the dual role of the ISR in medulloblastoma tumorigenesis. Moderate activation of the ISR promotes medulloblastoma formation, but strong activation of the ISR suppresses the tumor formation.

Apoptosis functions as a natural barrier to malignant transformation [37, 38]. During the course of cell transformation, apoptosis triggered by cellular stresses counters unrestrained cell proliferation driven by mutation of oncogenes. ER stress is one of the cellular stresses that are activated in cells during malignant transformation. Recent studies showed that PERK activation in response to ER stress either promotes cell transformation by decreasing apoptosis or suppresses the transformation by increasing apoptosis [12, 13, 14, 17]. A number of studies showed that the effects of the PERK-mediated ISR, beneficial or detrimental, on cells are activity-dependent [18, 39, 40, 41, 42]. The moderate ISR is beneficial to cells, but the strong ISR is detrimental to cells. Interestingly, our previous and current results indicate that the impact of the ISR on GCP apoptosis during cell transformation is also activity-dependent. Our previous paper showed that the decreased ISR via PERK heterozygous mutation enhances GCP apoptosis in hyperplastic lesions in young *Ptch1+/−* mice [27]. Herein, we showed that the strongly increased ISR via GADD34 homozygous mutation increased GCP apoptosis in hyperplastic lesions in young *Ptch1+/−* mice. Taken together, these data raise the possibility that the effects of the ISR, promoting or inhibiting, on tumorigenesis are determined by the activity of this pathway. Moderate activation of the ISR promotes cell transformation by suppressing apoptosis; however, strong activation of the ISR attenuates the transformation by facilitating apoptosis.

It was thought that strong activation of the ISR induces cell apoptosis through strong inhibition of protein biosynthesis and/or induction of CHOP [39, 43]. However, recent studies showed that CHOP induction is not necessary to lead to cell apoptosis [42, 44, 45]. We showed here that GADD34 homozygous mutation strongly increased the expression of CHOP in medulloblastoma but did not affect tumor cell apoptosis in adult *Ptch1+/−* mice. Moreover, we found that GADD34 homozygous mutation strongly enhanced the expression of ATF4 and increased pre-malignant GCP apoptosis in hyperplastic lesions in young *Ptch1+/−* mice. Nevertheless, IHC analysis showed that the immunoreactivity of CHOP was barely detectable in hyperplastic lesions in either young *Ptch1+/−; Gadd34+/+* mice or *Ptch1+/−; Gadd34−/−* mice (data not shown). Clearly, there is no co-relationship between strong CHOP induction and cell apoptosis in either hyperplastic lesions in young *Ptch1+/−; Gadd34−/−* mice or medulloblastoma in adult mice. These data rule out the possibility that CHOP is a major player in regulating cell apoptosis during the development of medulloblastoma in *Ptch1+/−; Gadd34−/−* mice. Therefore, it is possible that the strongly increased ISR induces GCP apoptosis in hyperplastic lesions in young *Ptch1+/−; Gadd34−/−* mice through strong inhibition of protein biosynthesis. Nevertheless, the molecular mechanisms by which the strongly increased ISR had no significant effect on medulloblastoma cells in adult *Ptch1+/−; Gadd34−/−* mice are unknown and merit further investigation.

While we found that the incidence of medulloblastoma in *Ptch1+/−; Gadd34+/−* mice was noticeably increased as compared to *Ptch1+/−; Gadd34+/+* mice (42.6% vs 30.3%, Figure 2B), the increase was not statistically significant. The conventional wisdom is that the value of the statistically non-significant data is in doubt. In this study, we used a large number of mice, around 70 mice per group. Moreover, the degree of increase (42.6%, *Ptch1+/−; Gadd34+/−* mice vs 30.3%, *Ptch1+/−; Gadd34+/+* mice) was quite impressive. It is likely that the low incidence of medulloblastoma in *Ptch1+/−* mice (30.3%) is responsible for the statistically non-significant result. Therefore, we believed that it was necessary to include these important data in this report. Although the number of hyperplastic lesions in young *Ptch1+/−; Gadd34+/−* mice was slightly increased as compared to *Ptch1+/−; Gadd34+/+* mice, we did not find evidence showing that GADD34 heterozygous mutation influenced pre-malignant GCP proliferation or apoptosis in the lesions. The mechanisms by which GADD34 heterozygous mutation noticeably enhances medulloblastoma tumorigenesis remain unknown. Clearly, the effects of GADD34 heterozygous mutation on medulloblastoma tumorigenesis warrant further investigation. A mouse model with a high incidence of medulloblastoma could be an ideal model to address this open question.

It has been shown that activation of the ISR in response to ER stress, which is induced by hypoxia and nutritional deficiency in the solid tumor microenvironment, plays a role in tumor development by regulating tumor cell viability, tumor invasion, and angiogenesis [9, 10, 11]. A report showed that GADD34 homozygous mutation enhances the ISR, increases VEGF-A expression and angiogenesis, and facilitates medulloblastoma development in mice that ectopically express the immune cytokine interferon-γ in the CNS during development [26]. Several studies also reported that the ISR stimulated the expression of VEGF-A in human medulloblastoma cells [46, 47]. Nevertheless, our previous study showed that PERK heterozygous deficiency does not influence cell proliferation or apoptosis, angiogenesis, or VEGF-A expression in medulloblastoma in adult *Ptch1+/−* mice [27]. In accordance with this previous study, we showed herein that GADD34 mutation, either heterozygous or homozygous, did not alter cell proliferation, cell apoptosis, angiogenesis, or VEGF-A expression in medulloblastoma in adult *Ptch1+/−* mice. Collectively, these data suggest that the effects of the ISR on tumor cells are cell-context dependent, possibly determined by genetic mutations and/or epigenetic changes in individual molecular subtypes of tumors.

We showed that GADD34 mutation, either heterozygous or homozygous, elevated the level of CHOP but did not change the level of p-eIF2α in medulloblastoma in adult *Ptch1+/−* mice. The elevation of p-eIF2α level is transient and highly dynamic in ER-stressed cells. Moreover, there is a lot of feedback in the ISR. The steady state levels of any ISR markers reflect the composite effect of the primary defect (such as GADD34 mutation) and the feedback response, which may over-shoot [8, 32]. Therefore, it is not surprising that we found the lack of effect of the GADD34 mutation on steady state levels of p-eIF2*α* in medulloblastoma in *Ptch1+/−* mice.

In summary, using GADD34 mutant mice, the results presented in this study advance our understanding of the effects on the PERK-mediated ISR on medulloblastoma development. Moderate increase of the ISR via GADD34 heterozygous mutation increased the incidence of medulloblastoma in *Ptch1+/−* mice. Nevertheless, strong increase of the ISR via GADD34 homozygous mutation decreased the incidence of medulloblastoma in *Ptch1+/−* mice by enhancing pre-malignant GCPs apoptosis during the course of cell transformation. This work represents the first *in vivo* experimental demonstration of the dual role of the ISR on tumorigenesis.

## MATERIALS AND METHODS

### Generation of mice

*Ptch1+/−* mice [21] on a mixed C57BL/6 × 129Sv background were purchased from Jackson laboratory (Bar Harbor, Maine). *Ptch1+/−* mice were crossed with *Gadd34−/−* mice on the C57BL/6 background [7, 26], and then the resulting progeny were intercrossed to obtain *Ptch1+/−*; *Gadd34+/+* mice, *Ptch1+/−*; *Gadd34+/−* mice as well as *Ptch1+/−; Gadd34−/−* mice. Genotypes were determined by PCR from DNA extracted from tail tips as described in previous papers [26, 27, 29]. Mice were monitored daily to detect medulloblastoma phenotypes, including ataxia, decreased movement, poor grooming, and doming of the skull, until the age of 8 months. All animal procedures were conducted in complete compliance with *the NIH Guide for the Care and Use of Laboratory Animals* and were approved by *the Institutional Animal Care and Use Committee* of the University of Minnesota.

### Histology, IHC, and TUNEL staining

Adult mice with medulloblastoma phenotypes and all 8-month-old asymptomatic mice received an intraperitoneal injection of 100 mg/kg BrdU (Sigma-Aldrich, St. Louis, MO) 2 h prior to perfusion. Six-week-old asymptomatic mice received a BrdU injection 8 h prior to perfusion. Anesthetized mice were perfused through the left cardiac ventricle with 4% paraformaldehyde in 0.1 M phosphate buffer. Brains were bisected in the sagittal plane, and one-half was postfixed for at least 48 h in 4% paraformaldehyde in PBS, dehydrated through graded alcohols, and embedded in paraffin. Serial sections of 5 μm thickness were cut and every tenth section in the series was routinely stained with H&E. The other half of the brain was postfixed for 1 h in 4% paraformaldehyde in PBS, cryopreserved in 30% sucrose for 48 h, embedded in OCT compound, and frozen on dry ice. Frozen sections were cut in a cryostat at 10 μm thickness. IHC for NeuN (1: 200, Millipore, Temecula, CA), PCNA (1:50,000, Sigma-Aldrich), GFAP (1:1000, Covance, Princeton, NJ), synaptophysin (1:200, Millipore), VEGF-A (1:50, Santa Cruz Biotechnology, Dallas, TX), and ATF4 (1:100, Abcam, Cambridge, MA) were performed on paraffin sections. IHC for CD31 (1:50, Santa Cruz Biotechnology) was performed on cryosections. All primary antibodies were detected using the Vectastain ABC kits (Vector Laboratories, Burlingame, CA) and 3′5′-diaminobenzidine/H_2_O_2_ reagent (Vector Laboratories) as substrate.

TUNEL staining was performed on paraffin sections using the ApopTag kit (Millipore) according to the manufacturer's instructions. IHC for BrdU (1:1000; Sigma-Aldrich), was performed on paraffin sections as described in our previous papers. We quantified positive cells for BrdU, TUNEL and VEGF-A as well as CD31 positive blood vessels in the center of medulloblastoma or hyperplastic lesions in the cerebellum as described in our previous papers [26, 27, 48].

### Western blot analysis

Cerebellar tissues were removed from adult control wild type mice as well as adult symptomatic *Ptch1+/−*; *Gadd34+/+* mice, *Ptch1+/−*; *Gadd34+/−* mice, and *Ptch1+/−; Gadd34 −/−* mice, which displayed typical medulloblastoma clinical phenotypes and enlarged cerebellum. These tissues were homogenized in 5 volumes of Triton X-100 buffer using a motorized homogenizer as previously described [27, 49]. After incubation on ice for 15 min, the extracts were cleared by centrifugation at 14,000 rpm for 30 min twice. The protein concentration of each extract was determined by DC Protein Assay (Bio-Rad Laboratories, Hercules, CA). The extracts (120 μg) were separated by SDS-PAGE and transferred to nitrocellulose membranes. The blots were then incubated with primary antibodies to p-eIF2α (1:1000, Cell Signaling Technology, Danvers, MA), eIF2α (1:1000, Santa Cruz Biotechnology), ATF4 (1:1000, Abcam), CHOP (1:1000, Thermo Scientific, Grand Island, NY), GFAP (1:1000, Covance), synaptophysin (1:1000, Millipore), and actin (1:5000, Sigma-Aldrich), followed by the HRP-conjugated secondary antibody (Vector Laboratories). The chemiluminescent signal was detected by the ECL Detection Reagents (GE Healthcare Biosciences, Piscataway, NJ). The intensity of the recorded chemiluminescence signal was quantified using the ImageQuantTL software from GE Healthcare Life Sciences.

### Statistical methods

Data were expressed as mean ± standard deviation (SD). Comparisons between multiple groups were statistically evaluated by the one-way ANOVA test using Prism 6 (GraphPad, Software Inc., La Jolla, CA). The incidence of symptomatic medulloblastoma between *Ptch1+/−; Gadd34+/+* mice and *Ptch1+/−; Gadd34+/−* mice as well as between *Ptch1+/−; Gadd34+/+* mice and *Ptch1+/−; Gadd34−/−* mice was statistically evaluated by Kaplan-Meier analysis and the incidence of total medulloblastoma between two groups was statistically evaluated by χ^2^ test using Prism 6 (GraphPad). *P* < 0.05 was considered significant.
